# Activation of MSRV-Type Endogenous Retroviruses during Infectious Mononucleosis and Epstein-Barr Virus Latency: The Missing Link with Multiple Sclerosis?

**DOI:** 10.1371/journal.pone.0078474

**Published:** 2013-11-13

**Authors:** Giuseppe Mameli, Giordano Madeddu, Alessandra Mei, Elena Uleri, Luciana Poddighe, Lucia G. Delogu, Ivana Maida, Sergio Babudieri, Caterina Serra, Roberto Manetti, Maria S. Mura, Antonina Dolei

**Affiliations:** 1 Department of Biomedical Sciences, University of Sassari, Sassari, Italy; 2 Department of Clinical and Experimental Medicine, University of Sassari, Sassari, Italy; 3 Department of Chemistry and Pharmacy, University of Sassari, Sassari, Italy; University of Liverpool, United Kingdom

## Abstract

The etiology of multiple sclerosis (MS) is still unclear. The immuno-pathogenic phenomena leading to neurodegeneration are thought to be triggered by environmental (viral?) factors operating on predisposing genetic backgrounds. Among the proposed co-factors are the Epstein Barr virus (EBV), and the potentially neuropathogenic HERV-W/MSRV/Syncytin-1 endogenous retroviruses. The ascertained links between EBV and MS are history of late primary infection, possibly leading to infectious mononucleosis (IM), and high titers of pre-onset IgG against EBV nuclear antigens (anti-EBNA IgG). During MS, there is no evidence of MS-specific EBV expression, while a continuous expression of HERV-Ws occurs, paralleling disease behaviour. We found repeatedly extracellular HERV-W/MSRV and MSRV-specific mRNA sequences in MS patients (in blood, spinal fluid, and brain samples), and MRSV presence/load strikingly paralleled MS stages and active/remission phases. Aim of the study was to verify whether HERV-W might be activated in vivo, in hospitalized young adults with IM symptoms, that were analyzed with respect to expression of HERV-W/MSRV transcripts and proteins. Healthy controls were either EBV-negative or latently EBV-infected with/without high titers of anti-EBNA-1 IgG. The results show that activation of HERV-W/MSRV occurs in blood mononuclear cells of IM patients (2Log10 increase of MSRV-type env mRNA accumulation with respect to EBV-negative controls). When healthy controls are stratified for previous EBV infection (high and low, or no anti-EBNA-1 IgG titers), a direct correlation occurs with MSRV mRNA accumulation. Flow cytometry data show increased percentages of cells exposing surface HERV-Wenv protein, that occur differently in specific cell subsets, and in acute disease and past infection. Thus, the data indicate that the two main links between EBV and MS (IM and high anti-EBNA-1-IgG titers) are paralleled by activation of the potentially neuropathogenic HERV-W/MSRV. These novel findings suggest HERV-W/MSRV activation as the missing link between EBV and MS, and may open new avenues of intervention.

## Introduction

Multiple sclerosis (MS) is a chronic neurological disease, which usually begins in early adulthood. It causes repeated unpredictable bouts of motor disorders, partial paralysis, sensory abnormalities and visual impairment, with demyelination and gliosis, various degrees of axonal pathology and episodic or progressive neurological disability. The aetiology is unknown and complex, but results from an inflammatory process that, among other effects, attacks and destroys oligodendrocytes, the cells that form the myelin sheaths around axons in the brain and spinal cord [1]. The immunopathogenic phenomena leading to MS are thought to be triggered by environmental (viral?) factors operating on a predisposing genetic background.

Several viruses have been proposed as co-factors that may contribute to MS risk [2]–[3], by direct (acute or persistent) infection of the brain, or peripheral infection activating cross-reactive T-cells, acting against nerve myelin [4]. The most consistent studies (and confirmed by independent groups) for a potential virus involvement in MS exist for the Epstein Barr virus (EBV) [5]–[8], and for two members of the W family of human endogenous retroviruses (HERV-W): MSRV (MS associated retrovirus), and ERVW-1, an element expressing only the env protein, named Syncytin-1, as reviewed in [9]–[13].

The MSRV element (MS-associated retrovirus) is the first known member of the HERV-W family [14]; it has been detected and purified from cells of MS patients, as free virus-like particles, carrying RT activity and an RNA genome with terminal repeats, *gag*, *pol* and *env* regions. The other HERV-W member related to MS is an element located on chromosome 7q21–22, that has inactivating mutations in the *gag* and *pol* genes and is not able to form virus-like particles [15]. The env product of ERVW-1 has been named syncytin-1, since it is produced by placental trophoblasts and causes their fusion, to form the syncytial layer during pregnancy [16]. The syncytin-1 protein can be found intracellularly and on the plasma membrane, but has not been detected extracellularly, nor its RNA sequence in virus-like particles [10].

Independent studies have shown that MSRVenv and syncytin-1 proteins share several potentially pathogenic, biological features, as induction of T mediated immunopathology, of pro-inflammatory cytokines and T-cell responses, with polyclonal expansion, as reviewed in [10]–[11], and have been shown to cause neurotoxic effects *in vitro* and in humanized or transgenic animal models [15], [17]: they may cause neuroinflammation, neurodegeneration, alterations of the immune system and stress responses; both have been suggested as co-factors triggering the immuno-pathogenesis of MS. Expression of HERV-W/MSRV/syncytin-1 occurs in astrocytes of MS lesions of the brain [15], [18]–[20], as well as in endothelial and microglial cells [21]. In a mouse model, oligodendrocytes (which produce the myelin sheath of the nerve) were shown to be sensitive to syncytin-mediated release of redox reactants from astrocytes [15].

Studies from our group found repeatedly retrovirus-like MSRV particles and MSRV-specific mRNA sequences in MS patients (in blood, spinal fluid, and brain samples), and MRSV presence/load strikingly paralleled disease behaviour. A multicentre study of MS patients and controls from different European areas showed that MSRV presence and load in blood and spinal fluid was significantly associated to MS in all ethnic groups [22]. Direct parallelisms were observed between MSRV positivity/load (in blood, spinal fluid, and brain samples) and MS temporal and clinical stages, as well as active/remission phases; moreover MSRV positivity of spinal fluids increased with MS duration [23]. Notably, MSRV presence in spinal fluids of early MS cases was related to worse prognosis in the next ten years: starting from comparable conditions, after three [24], six [25], and ten years [26], mean disability, annual relapse rate, therapy requirement and progression to secondary-progressive MS were significantly higher in patients starting with MSRV-positive spinal fluids. When we monitored MS patients receiving treatment, a major reduction of HERV-W/MSRV/syncytin-1 was detected in patients undergoing efficacious, current therapies [13], [27]. Notably, in a longitudinal evaluation, a patient, after initial clinical and virological benefit, had MSRV rescue, *preceding* strong disease progression and therapy failure [27]. From the above findings, we proposed that evaluation of plasmatic MSRV could be considered the first prognostic marker for the individual patient, to monitor disease progression and therapy outcome. This possibility is reinforced by the study of patients with optic neuritis, a disease frequently prodromic to MS: MSRV positivity of patients was found significantly higher than that of pathological controls, and the conversion to full-blown MS in the next 20 months occurred only among MSRV-positive patients [28]. In line with our findings, an independent 1-year follow up study of MS patients observed significant decreases in anti-HERV-Wenv and anti-HERV-Henv antibody reactivity as a consequence of IFN-β therapy, closely linked to efficacy of therapy/low disease activity [29]. Other HERVs were reported to be activated in MS patients [3], [9], [12]. However none of them, but HERV-W, has been shown to exert potentially (neuro)pathogenic properties.

As for EBV, seroepidemiological studies have provided substantial support for the association between EBV and MS [5]–[8], [30]–[31]. The risk of MS is low in EBV-negative individuals [32]–[34], and increases several folds following EBV infection, particularly if the EBV infection occurs in late adolescence or adulthood, when the infection is symptomatic. Indeed, in 35 to 50 per cent of the cases, the delayed EBV primary infection causes infectious mononucleosis (IM), that has been associated with a two-to-four fold increased risk of MS, [5], [35]–[38]. There is also an increased risk of MS among EBV-positive children [39]. A meta-analysis showed an association between the appearance of anti-EBV antibodies and the MS onset, 5–20 years later [7]; another one reported that the relative risk of MS for a past history of IM is 2.17 [8]. In addition, elevated serum titers of pre-onset anti-EBNA antibodies have been associated with an increased risk of MS [35], [40]. A recent meta-analysis of all data published from from 1960 to 2012 showed a significant OR for sero-positivity to anti-EBNA (EBV nuclear antigens) IgG and anti-VCA IgG in MS cases, but not for anti-EA IgG sero-positivity [6]. Notably, high levels of anti-EBNA IgG antibody are established risk factors for MS [35].

There is convincing epidemiological evidence that late EBV primary infection/seroconversion is a risk factor for MS development, nevertheless, the relationship between EBV and MS pathogenesis remains elusive [4], [30]–[31]. No studies have found yet evidence of EBV expression specific to MS, and currently there is no consensus in establishing whether there is a presence of EBV-infected cells in the central nervous system (CNS) of MS patients[30], [41]–[42]. Therefore, it remains to be determined whether EBV would continue to play a role after disease initiation.

Several potentially pathogenic mechanisms explaining an EBV causative role for MS, yet very speculative, have been proposed, as recently reviewed [4], [30]; they include molecular mimicry (generation of autoreactive T cells are primed against EBV, but cross-reacting with CNS antigens), and generation of EBV-infected autoreactive B cells (that would seed the target organ expressing the autoantigen and produce autoantibodies and costimulatory signals to autoreactive T cells).

Recently, we showed that the binding to the plasmamembrane of EBVgp350, the major envelope protein of EBV, is sufficient to activate the potentially immunopathogenic and neuropathogenic HERV-W/MSRV/syncytin-1, in cells that may be involved in MS pathogenesis, deriving from blood and brain [13]. Hence, we made the hypothesis of a possible involvement of both viruses in MS pathogenesis, with the possibility for MSRV of a direct role of effector of pathogenicity, and for EBV of an initial trigger of future MS, years later. This “dual virus hypothesis” has been done also for torque teno virus [43], but an association between infection by this ubiquitous virus and MS could not be established so far [44].

Aim of the present study was to verify whether HERV-W activation by EBV might occur also *in vivo*, during a delayed primary infection by EBV, in young adults with IM symptoms requiring hospitalization. Indeed, the data indicate that the main links between EBV and MS (IM and high anti-EBNA-IgG titers) are paralleled by activation of the potentially neuropathogenic HERV-W/MSRV.

## Materials and Methods

### Ethics Statement

The study was approved by the Sassari ASL Ethics Committee (Protocol number 1120/L). The patients and the volunteers gave written informed consent. Written informed consent from the next of kin (which was one of the parents in all cases) on the behalf of minors (under the age of 18) was obtained.

### Patients

Seventeen consecutive IM patients (mean age 23.2±5.7 years, median 22, 13 females and 4 males), hospitalized in the Section of Infectious Diseases, Department of Clinical and Experimental Medicine, University of Sassari, were enrolled. Laboratory diagnosis of acute EBV infection was made by serological assays confirming the presence of EBV viral capsid antigen (VCA) IgM antibodies. For each patient, the data collected included type and duration of symptoms, family history for autoimmune diseases, including MS, neurological signs, routine IM-related clinical symptoms and signs, hematological values, and any steroid therapy. Serum EBV-specific IgG and IgM antibodies were detected by commercially available Chemiluminescence Immunoassays (CLIA), following manifacturer’s instructions (LIAISON® VCA IgG, IgM and EBNA-1 IgG, DiaSorin S.p.A. Vercelli, Italy). In most patients EBV DNA was evaluated in the plasma by real-time PCR (EBV ELITe MGB™ Kit, according to the manufacturer’s protocols. Twenty four matched healthy donors (HD, mean age 26.2±4.8 years, median 25, 16 females and 8 males) were included as controls. Nine of them were EBV-negative, while fifteen had been infected by EBV previously. Of the latters, eight individuals had high levels of IgG antibody to the Epstein-Barr nuclear antigens (EBNA-1 IgG, titers >600 IU/ml). On Table 1 the most relevant demographic and clinical characteristics of hospitalized IM patients and of healthy controls are reported.

**Table 1:** Demographic and clinical characteristics of patients hospitalized with infectious mononucleosis and of healthy controls.

Sample	Age	Sex	Days from onset	Fever(°C)	VCA IgM(UI/ml)	VCA IgG(UI/ml)	EBNA-1 IgG(UI/ml)	EAIgG(UI/ml)	EBVDNA^a^
**IM-1**	18	M	5	38.5	>160	80	neg	115	<185
**IM-2**	23	F	8	40	>160	126	neg	bl^b^	neg
**IM-3**	19	F	9	38.5	106	neg	neg	neg	nd^c^
**IM-4**	20	F	10	39	>160	146	neg	>150	nd
**IM-5**	23	F	10	37.5	>160	35	neg	bl	neg
**IM-6**	32	F	10	38	>160	33	bl	neg	5,725
**IM-7**	22	F	10	37.5	83	119	neg	104	nd
**IM-8**	22	F	12	39	>160	195	bl	69	neg
**IM-9**	23	M	12	39	115	231	neg	>150	1,685
**IM-10**	23	F	15	38.5	49	21	neg	neg	225
**IM-11**	21	F	16	38	>160	58	bl	65	<185
**IM-12**	14	F	17	39	>160	108	neg	>150	180
**IM-13**	22	F	18	38	Pos	pos	pos	bl	nd
**IM-14**	27	M	19	38.5	>160	58	neg	54	401
**IM-15**	19	F	23	38.2	>160	neg	neg	neg	nd
**IM-16**	40	M	37	39.5	95	neg	neg	neg	neg
**IM-17**	27	F	55	37.5	>160	345	>600	>150	neg
**HD-1**	20	F	–	–	Neg	neg	neg	neg	nd
**HD-2**	21	F	–	–	Neg	neg	neg	neg	nd
**HD-3**	22	F	–	–	Neg	neg	neg	neg	nd
**HD-4**	23	F	–	–	Neg	neg	neg	neg	nd
**HD-5**	23	F	–	–	Neg	neg	neg	neg	nd
**HD-6**	27	F	–	–	Neg	neg	neg	neg	nd
**HD-7**	31	F	–	–	Neg	neg	neg	neg	nd
**HD-8**	31	M	–	–	Neg	neg	neg	neg	nd
**HD-9**	32	F	–	–	Neg	neg	neg	neg	nd
**HD-10**	20	M	–	–	Neg	483	157	neg	nd
**HD-11**	21	M	–	–	Neg	137	246	neg	nd
**HD-12**	23	F	–	–	Neg	158	48	nd	nd
**HD-13**	23	F	–	–	Neg	143	142	neg	nd
**HD-14**	26	M	–	–	Neg	nd	<600	nd	nd
**HD-15**	28	M	–	–	Neg	81,3	289	neg	nd
**HD-16**	29	F	–	–	Neg	nd	<600	nd	nd
**HD-17**	23	F	–	–	Neg	nd	>600	nd	nd
**HD-18**	23	F	–	–	Neg	nd	>600	nd	nd
**HD-19**	25	F	–	–	Neg	>750	>600	neg	nd
**HD-20**	26	M	–	–	Neg	>750	>600	neg	nd
**HD-21**	29	F	–	–	Neg	>750	>600	neg	nd
**HD-22**	29	M	–	–	Neg	176	>600	neg	nd
**HD-23**	35	F	–	–	Neg	neg	>600	neg	nd
**HD-24**	39	M	–	–	Neg	nd	>600	nd	nd

a data are expressed as EBNA-1 DNA copies/ml of plasma. Detection limits: 100 copies/ml of plasma;

b bl, borderline;

c not done.

### Blood Cell Separation

Peripheral blood mononuclear cells (PBMC) were isolated from heparinized peripheral blood samples by layering on Ficoll/Hipaque, as described [27]. Cell viability was assessed with the Trypan blue exclusion method. The cells were either frozen in deep cold, or processed immediately for further studies (see below).

### RNA Extraction and Real-time RT-PCR

The polyA+ RNAs were extracted from 50.000 cells, by mRNA Dynabeads® kit (Dynal Biotech, Oslo, NO) and retrotranscribed, as described [13]. Selective amplification of the MSRV-type HERV-W*env* transcripts was obtained by a discriminatory real time RT-PCR assay, as published in [45]. The RNA amounts used for retrotranscription were 1.5 µg/sample, and the PCR temperature was 60°C. The quantification of cDNA sequences was obtained by external calibration curves, obtained from serial dilutions of plasmids containing the amplicon of interest (10–10^5^ copies/well), as described previously [18]. The TaqMan® MSRV*env* probe was designed by the Beacon Designer software (PREMIER Biosoft International CorinaWay Palo Alto, CA). To control for correct amplification and retrotranscription, a positive sample was also submitted to each RNA extraction procedure, and the resulting extract was amplified in triplicate. Parallel RNA samples were also exposed to PCR amplification without the RT step, to detect contaminant DNA. Prevention measures against cross-contamination were employed. For each sample, the Ct (cycle threshold) value of the gene of interest (GI) was normalized by comparison to the Ct of the glyceraldehyde-3-phosphate dehydrogenase (GAPDH) invariate housekeeping gene, assuming that: (i) a difference of 1 between sample Cts means that the sample with the lower Ct value had double the target sequence of the other sample, and (ii) the rate of Ct change versus the rate of target copy change is identical for the gene of interest and the housekeeping gene. housekeeping gene. The data have been expressed according to the 2^−ΔCt^ Method, according to the following formula:
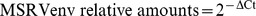
where



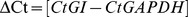



### Flow Cytometry Analyses

Aliquots of 250,000 alive PBMC were employed for each evaluation. The detection of HERV-Wenv protein on the plasma membrane, as well as the phenotype of PBMC subpopulations, were performed by flow cytometry as described [13]. Briefly, the detection of HERV-Wenv protein on the plasma membrane was performed using anti-HERV-Wenv rabbit polyclonal antibody (Allele Biotec, 2 µg/sample, for 20 minutes at room temperature in the dark) and secondary fluorescein isothiocyanate-conjugated goat anti-rabbit IgG (Sigma-Aldrich). The isotype control was a pre-immune rabbit serum (Santa Cruz Biotechnology, Inc). To determine the phenotype of cell subpopulations, the PBMC were stained for 20 minutes at room temperature in the dark with allophycocyanin-conjugate anti-CD14, phycoerythrin-conjugated anti-CD19, and peridinin-chlorophyll-conjugated anti-CD16 antibodies (BD Biosciences). As isotype control, an anti-rabbit IgG antibody was used (Santa Cruz Biotechnology, Inc). Cells were analysed on a FACS Calibur flow cytometer, using Cells Quest software (Becton-Dickinson). The area of positivity was determined using an isotype-matched antibody; a total of at least 50,000 events for each sample were acquired.

### Statistical Analysis

Descriptive statistics included mean, median, standard deviation (SD), interquartile range. The Shapiro-Wilks test was used to check the normality of the distribution. Between-group differences were assessed by using Kruskall Wallis and Mann–Whitney U-test for continuous nonnormally distributed variables, as appropriate. The relationship between nonparametric variables were evaluated calculating the Spearman’s correlation coefficient (rho) and its significance.

## Results

### Patient Characteristics

Blood samples of patients hospitalized with laboratory-confirmed diagnosis of IM were collected during the acute phase. Mean disease duration at the time of blood collection was 16.8±11.9 days from onset of symptoms, median 12 days, range 5–55 days. Eightytwo percent of the patients was in the first two weeks from IM onset, when the major immunologic and virological changes during primary EBV infection occur [46]. None of the patients or healthy controls had recognized risk of MS. The main characteristics of the study participants are reported on Table 1.

### Expression of MSRV*env* Transcripts

**Figure 1:**
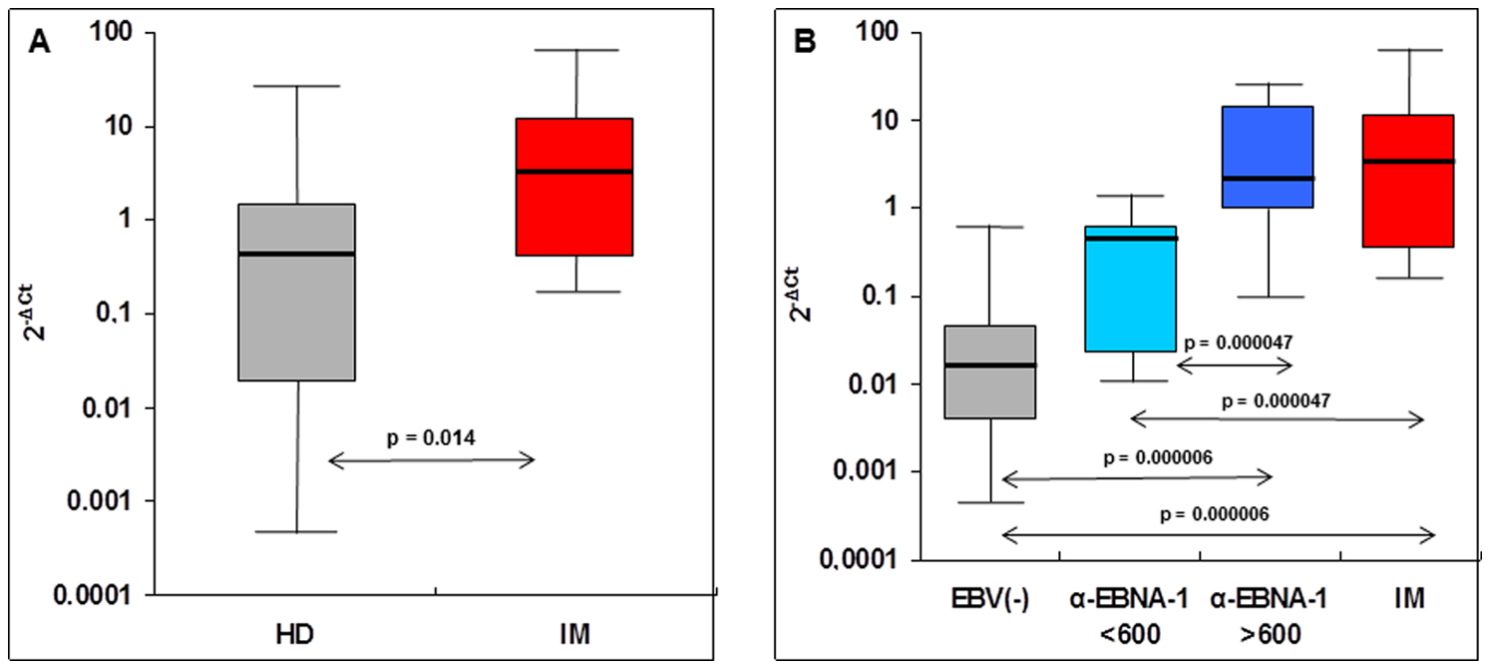
MSRV-type mRNA expression by PBMC from IM patients and healthy donors (HD). The amounts of MSRV-type HERV-Wenv transcripts were evaluated by discriminatory real time RT-PCR, and normalized by the 2^−ΔCt^ method (see Methods for details). Data are expressed as medians (line), with maximum and minimum values (whiskers); boxes represent interquartile range of the samples. Statistical significance was evaluated by the Mann-Whitney U-test for comparison of two groups, and by the Kruskal-Wallis test for three or four groups. (**A**) Comparison of IM patients (N = 17) and all healthy donors (HD, N = 24). (**B**) Comparison of IM patients (N = 17) and HD donors stratified according to plasmatic anti-EBNA-1 IgG titers, as EBV-negative (EBV−, N = 9), <600 IU/ml (N = 7), and >600 IU/ml (N = 8). Kruskall-Wallis test gave p = 0.0005 for all four groups, and p = 0.014 for the three HD groups.

The relative quantification of MSRVenv transcripts in PBMC of patients and controls was carried out by MSRVtype-specific real-time RT-PCR [45]. Normalysed data are reported on Figure 1: there is a statistically significant increase of MSRV*env* transcripts in whole PBMC from IM patients with respect to healthy subjects (the median of IM patients is 6.9 fold higher than the one of healthy donors, p = 0.014).

Since high titers of anti-EBNA antibodies have been associated with increased risk of MS [35], [40], the healthy donors were stratified according to their anti-EBNA-1 IgG titers, as uninfected (negative), or previously infected with low or high anti-EBNA-1 IgG titers (below or above 600 IU/ml of plasma, respectively). The data reported on Figure 1B indicate that the amounts of MSRVenv mRNA present in PBMC of healthy individuals are strictly related to those of anti-EBNA-1 IgG (p = 0.0005). However, the highest MSRVenv mRNA values are observed in the blood of IM patients, i.e. during the acute, highly symptomatic phase of the primary infection. In facts, the MSRVenv mRNA median value of IM patients is 194-fold higher that of uninfected persons; the values of persons with high and low anti-EBNA-1 IgG titers are 131- and 28-fold, respectively, the MSRVenv mRNA levels of uninfected persons, with high statistical significance.

### Presence of the HERV-Wenv Protein on the Plasma Membrane

**Figure 2:**
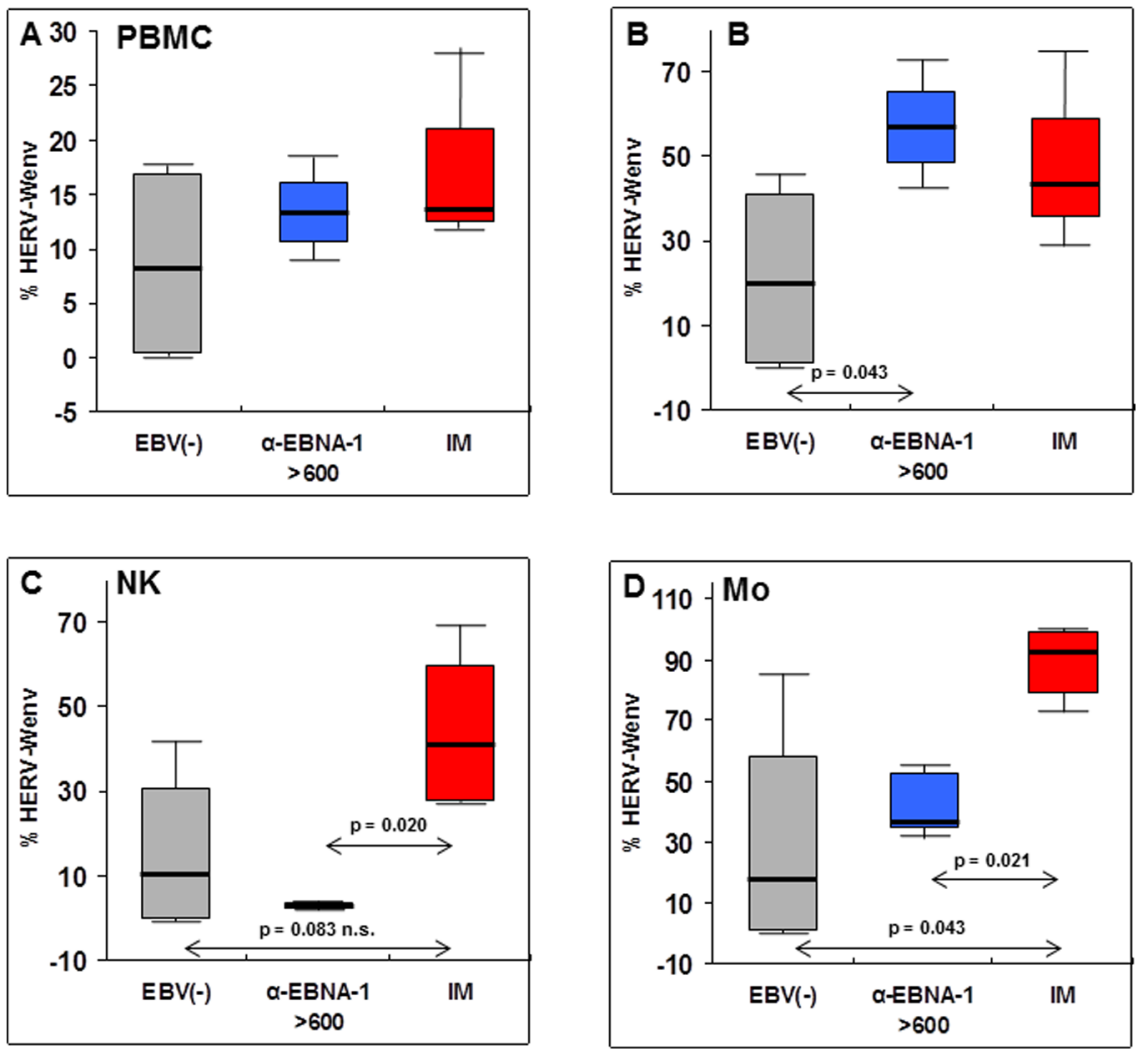
Presence of HERV-Wenv protein on the plasma membrane of PBMC subpopulations from IM patients and controls. Flow cytometry evaluation of alive PBMC from IM patients (IM) and healthy donors (HD), either with anti-EBNA-1 IgG titers >600 IU/ml or EBV− negative (EBV−). Data were evaluated as percentage of cells with HERV-Wenv-specific staining, as specified in Methods, and are expressed as medians (line), with maximum and minimum values (whiskers); boxes represent interquartile range of the samples. Statistical significance was evaluated by the Mann-Whitney U test for comparison of two groups. When not specified, differences were not significant. All samples (four for each group) were evaluated in the same day. (**A**) Comparison of HERV-W-specific staining of whole PBMC. (**B**), (**C**), (**D**) HERV-W-specific staining of PBMC subpopulations sorted by specific staining with anti-CD19, anti-CD16 and anti-CD14 antibodies, respectively.

Due to the high percentage of identities between the HERV-Wenv genes, no antibody specific for a unique HERV-Wenv protein has been identified so far [13]. Therefore it cannot be discriminated whether an env protein is a MSRV-type env or syncytin-1, and data must be expressed as HERV-Wenv positivity only. Alive PBMC of four IM patients were analyzed by flow cytometry in comparison to four EBV-negative healthy individuals and four persons with past (latent) EBV infection and high anti-EBNA-1 IgG titers, with respect to the presence of HERV-Wenv protein on the outer surface of the plasma membrane. The analysis was limited to four individuals for each group, in order to compare only values collected in the same session, to avoid technical variations. The results are reported on Figure 2. As shown on Figure 2A, in whole PBMC the HERV-Wenv positivity of IM patients and of persons with past EBV infection is almost twice that of EBV-negative subjects. The HERV-Wenv protein, however, is not uniformly exposed by all the PBMC, but derives from an uneven expression in the various cell subsets. No statistical significance is reached in Figure 2A, since the main component of PBMC is the T cell compartment, that was previously shown to be totally negative for HERV-Wenv expression, both at the protein and at the RNA level [13], [47]; therefore it was not analysed specifically. However, no HERV-Wenv expression was detected on the plasma membrane of CD19−/CD16−/CD14− cells, that comprises the T cells (data not shown). As shown in Panels 2B–D, in uninfected individuals the median HERV-Wenv positivity of CD19+, CD16+ and CD14+ cells (B lymphocytes, NK cells and monocytes, respectively) is around 19, 10 and 17%, respectively. The env positivity of the CD19+ B cell compartment (Figure 2B) is more than doubled after EBV infection (IM patients: 43%, latently infected highly anti-EBNA-1 positive individuals: 56%, p = 0.043). As for the CD16+ NK cell subset (Figure 2C), there is a 4-fold increase in HERV-Wenv expression during IM (40%, p = 0.020), but a deep reduction in latently infected highly anti-EBNA-1 positive individuals: 3%). The CD14+ monocyte compartment (Figure 2D) showed the highest increases of HERV-Wenv expression: 5.4-fold increase during IM (mean positivity: 92%, p = 0.043) and 2.6-fold increase during highly anti-EBNA-1 positive latency (44%); also the difference between the latter two group reaches the statistical difference (p = 0.021).

## Discussion

A history of IM and high anti-EBV titers are factors that increase the risk of MS [5], [7]–[8], [35]–[38], [40]. The mechanism by which EBV infection is linked to MS pathogenesis is still unknown [4], [30]–[31]. No studies support unequivocally a direct etiologic role of EBV or prove its preferential presence, expression or reactivation in MS patients with respect to controls to MS, and currently there is no consensus in establishing whether there is a presence of EBV-infected cells in the CNS of MS patients[30], [41]–[42]. Therefore, it remains to be determined whether EBV would continue to play a role after disease initiation.

On the other hand, potentially neuropathogenic endogenous retroviruses of the W family are expressed in the blood and in the brain of MS patients in close parallelism with disease progression [23], [26]–[27]. Recently we have found a link between EBV and HERV-W, since we showed that the binding *in vitro* to the plasmamembrane of the EBVgp350 major envelope protein is able to activate the expression of the potentially immunopathogenic and neuropathogenic HERV-Ws in in cells that may be involved in MS pathogenesis (B cells, monocytes, macrophages and astrocytes) [13]. Hence, we made the dual virus hypothesis of a possible involvement of both viruses in MS pathogenesis, with the possibility for MSRV of a direct role of effector of pathogenicity, and for EBV of an initial trigger of future MS, years later.

In the present report, we provide for the first time data showing that activation of HERV-W/MSRV occurs *in vivo* in patients affected by IM. With respect to EBV-negative healthy controls, the increase of MSRV-type env mRNAs in IM patients is almost 2 Log_10_ (p = 0.000006, Figure 1B). More interestingly, when healthy controls are stratified with respect to previous EBV infection (high and low, or no anti-EBNA-1 IgG titers), a close correlation occurs for MSRV mRNA activation (Sperman rho = 0.60, p<0.05).

These novel findings are reinforced by the flow cytometry data on the activation also of the HERV-Wenv protein product, reported in Figure 2 as the percentage of PBMC expressing the HERV-Wenv protein on the membrane. The HERV-W activation is not uniform in all cell subtypes nor in acute disease and past infection, i.e. the latency state (Figures 2B–D). With respect to uninfected healthy individuals, IM patients showed a doubling of HERV-W positivity of B cells, and 4-fold and 5.5-fold increases in NK cells and monocytes, respectively. In past infection with high anti-EBNA-1 IgG titers, B cells continue to express similar HERV-Wenv levels, while the effect is reduced on monocytes (2.6-fold higher than in uninfected individuals), and NK cells became almost HERV-Wenv-negative. We have no explanation for the latter finding. On the whole, the data indicate that post-transcriptional regulation of HERV-Wenv occurs differently in specific cell subsets, in keeping with our previous observations [13].

Thus in our hands both ascertained links between EBV and risk of future MS (IM and high anti-EBNA-1 titers) do stimulate the expression of HERV-W/MSRV.

As for the mechanisms of HERV-W activation, we showed recently that *in vitro* EBV activates HERV-W/MSRV/syncytin-1, in cells from blood and brain, that might be involved in the pathogenesis of MS [13]. There is no need of EBV entry or expression, since the effect was seen also after exposure to the EBVgp350 major envelope protein. In EBVgp350-treated PBMC, MSRV*env* and syncytin-1 transcription was activated in B cells and monocytes, but not in T cells (that do not express HERV-W genes), nor in the highly positive NK cells. The latter cells (as the monocytes and the B, but not the T cells) were activated by proinflammatory cytokines, likely with involvement of the protein kinase C signalling enzyme [13]. The effect of EBVgp350 was abolished by silencing NF-κB, a transcription factor that is implicated in the production, by activated astrocytes, of pro-inflammatory cytokines: this, in turn, induces further release of cytokines, that contribute *in vivo* to exacerbating the neurodegenerative process [48], and that *in vitro* stimulate HERV-W*env* expression in astrocytes (unpublished data) and PBMC [13], [49]. It is likely that *in vivo*, during EBV replication in IM patients, the EBVgp350 protein could activate HERV-W/MSRV expression, as we showed *in vitro* [13]. In addition, a cytokine-mediated mechanism could cooperate with EBV for HERV-W activation. As known, several cytokines are produced during acute IM. In a prospective study of late primary EBV infections, Balfour *et al.* [46], detected elevated levels of eight cytokines that might contribute to IM pathogenesis, among which interferon, TNFα, and IL-6 the latter was correlated significantly with IM severity and high EBV viremia. Of note, IL-6 is a major cytokine in the central nervous system, that has been related to many brain diseases, among which MS [50]. The above cytokines do increase HERV-W expression/release *in vitro* [13], [49], and are increased *in vivo* during MS exacerbations [50].

Of particular interest is the effect of EBV activation of HERV-W/MSRV in the vast majority of monocytes during the acute infection (Figure 2D, IM), for possible involvements in MS pathogenesis. *In vitro*, these cells were shown to be the most responsive to EBVgp350, reaching, after exposure to the EBV protein, HERV-W levels higher than those detected in B cells, particularly after cell differentiation into macrophages [13]. In MS and other neurological diseases, significant infiltration of immune cells from the periphery into the CNS (and activation of microglia) is largely observed, and it accounts for the increased representation of macrophages within the CNS; the functions of activated macrophages (and microglia) within the CNS are complex, as they are implicated in both demyelination and remyelination of MS lesions, as observed in animal models [51]. Increased blood-brain barrier permeability is a characteristic hallmark of the CNS alterations leading to MS, suggesting a causal relationship between inflammatory cell recruitment into the CNS and the blood–brain barrier dysfunction [52]. Activated monocytes easily pass across the endothelia, and could carry the HERV-W across the blood-brain barrier, entering the brain, where the HERV-Wenv protein could contribute to the pathogenicity, inducing inflammation, demyelination and axonal damage [6], [10]–[13]. Thus, the HERV-W expression (and release) by monocyte-macrophages can reasonably account for the bulk of expression (and effects) of these elements. Also the high levels of HERV-W expression in B cells throughout the IM and the post-infection stages (Figure 2B), as well as in MS patients [13] might contribute to the process, since B cells have been proposed to be relevant to the etiology and pathogenesis of MS [53], and MS therapies targeting B cells seem effective against the relapsing-remitting forms [54].

As for the post-infection stages of EBV latency, it is well known that during latency, EBV expresses a limited repertoire of proteins, non-coding RNAs and microRNAs [54]. The virus remains immunologically silent in small numbers of memory B cells, which traffic into inflamed tissues, and are tightly controlled by invariant natural killer T cells [55] and by cytotoxic T lymphocyte cells to prevent lymphoproliferation [56]. Since HERV-W is expressed by a high proportion of B cells (Figure 2B), it is likely that reciprocal interactions occur within these cells during latency, that could play an additional role to the indirect mechanism proposed by Meier and coworkers, by which latent EBV infection could contribute to (neuro)inflammation, through the expression of small non-coding RNAs that bind to Toll-like receptor 3 and potentially other intracellular receptors [56]. Notably, EBNA-1 is the only viral protein commonly expressed in latency programs that allow the persistence of EBV in B-cells during activation and differentiation and in a variety of other cells types, and this protein has been shown to reprogram cellular transcription and signaling [58]–[59]. Moreover, its binding to several cellular promoters has been documented [60]. Therefore a contribute of EBNA-1 to HERV-W activation in these cells could be hypothesized, either by direct HERV-W activation in circulating B cells, or indirectly, through induction of HERV-W-activating stimuli. A major issue of the present paper is not only the finding that HERV-W is activated during IM, but also that it is observed in a subset of healthy, latently-infected individuals (those with antiEBNA-1 titers >600). A long prospective study showed that elevations of antibody titers to the EBNA complex and EBNA-1 among MS cases first occurred between 15 to 20 years before the onset of symptoms and persisted thereafter [57]. We show here that high HERV-W expression occurs in healthy persons with high anti-EBNA Ig titers, thus concomitantly to what has been considered among the early events in the pathological process that leads to MS, perhaps in individuals presenting a predisposing genetic background.

In conclusion, our data indicate that the two main links of EBV to MS (past IM and high anti-EBNA-1 IgG titers) are associated to the activation of potentially neuropathogenic HERV-W/MSRV, both at the mRNA and the protein levels. These novel findings reinforce our hypothesis of a possible involvement of both viruses in MS pathogenesis [13], with the possibility for MSRV of a direct role of effector of pathogenicity, and for EBV of an initial trigger of future MS, years later, and indicate the activation of HERV-W/MSRV as the possible missing link between EBV and MS. These findings may open new avenues of intervention against MS.
